# Defining Optimal Head-Tilt Position of Resuscitation in Neonates and Young Infants Using Magnetic Resonance Imaging Data

**DOI:** 10.1371/journal.pone.0151789

**Published:** 2016-03-22

**Authors:** Utpal S. Bhalala, Malvi Hemani, Meehir Shah, Barbara Kim, Brian Gu, Angelo Cruz, Priya Arunachalam, Elli Tian, Christine Yu, Joshua Punnoose, Steven Chen, Christopher Petrillo, Alisa Brown, Karina Munoz, Grant Kitchen, Taylor Lam, Thangamadhan Bosemani, Thierry A. G. M. Huisman, Robert H. Allen, Soumyadipta Acharya

**Affiliations:** 1 Department of Anesthesiology and Critical Care Medicine, The Johns Hopkins University, Baltimore, Maryland, United States of America; 2 Center for Biomedical Innovations and Design, Department of Biomedical Engineering, The Johns Hopkins University, Baltimore, Maryland, United States of America; 3 Division of Pediatric Radiology and Pediatric Neuroradiology, Department of Radiology and Radiological Science, The Johns Hopkins University, Baltimore, Maryland, United States of America; Centre Hospitalier Universitaire Vaudois, FRANCE

## Abstract

Head-tilt maneuver assists with achieving airway patency during resuscitation. However, the relationship between angle of head-tilt and airway patency has not been defined. Our objective was to define an optimal head-tilt position for airway patency in neonates (age: 0–28 days) and young infants (age: 29 days–4 months). We performed a retrospective study of head and neck magnetic resonance imaging (MRI) of neonates and infants to define the angle of head-tilt for airway patency. We excluded those with an artificial airway or an airway malformation. We defined head-tilt angle a priori as the angle between occipito-ophisthion line and ophisthion-C7 spinous process line on the sagittal MR images. We evaluated medical records for Hypoxic Ischemic Encephalopathy (HIE) and exposure to sedation during MRI. We analyzed MRI of head and neck regions of 63 children (53 neonates and 10 young infants). Of these 63 children, 17 had evidence of airway obstruction and 46 had a patent airway on MRI. Also, 16/63 had underlying HIE and 47/63 newborn infants had exposure to sedative medications during MRI. In spontaneously breathing and neurologically depressed newborn infants, the head-tilt angle (median ± SD) associated with patent airway (125.3° ± 11.9°) was significantly different from that of blocked airway (108.2° ± 17.1°) (Mann Whitney U-test, p = 0.0045). The logistic regression analysis showed that the proportion of patent airways progressively increased with an increasing head-tilt angle, with > 95% probability of a patent airway at head-tilt angle 144–150°.

## Introduction

Birth asphyxia is responsible for an estimated 717,000 newborn deaths every year, or about 23% of the global burden of newborn deaths [1]. Effective newborn resuscitation is essential in reducing the sequelae of birth asphyxia [2,3]. Resuscitation programs recommend that for those newborn who do not start breathing despite drying and stimulation, positive-pressure ventilation should be initiated within one minute after birth [4]. One of the challenges of positive-pressure ventilation in unconscious or sedated children is a tendency for airway obstruction due to relaxation of airway tone and glossoptosis [5,6]. The head-tilt maneuver for airway patency involves extension of the head at the atlanto-occipital joint and, coupled with the chin-lift maneuver, is a well-described airway maneuver for airway patency during resuscitation [6,7,8]. Though there is well-documented literature on the relationship of the chin-lift maneuver with airway patency, the relationship between the angle of head-tilt and airway patency has not been defined [9]. There are conflicting results of studies on the relationship of head extension and airway patency in adults [6,10,11]. For newborns, the Neonatal Resuscitation Program (NRP) recommends “sniffing position” (neck flexion with upper cervical extension) for airway patency using a roll under the neck to compensate for the large occiput [4]. The Neonatal Life Support (NLS) program recommends positioning the child’s head in a neutral position using a roll under the shoulder to achieve upper airway patency. However, for both newborns and infants, neither the sniffing position nor the neutral head position has been evaluated for airway patency [12]. Also, an improper placement of a roll under the neck or shoulder could potentially jeopardize the airway. Defining angle of head-tilt for airway patency would help clarify the current controversy of the two guidelines related to the head-neck position during neonatal resuscitation. The information may also be used to create a neonatal resuscitation mat with a built-in shoulder-roll to ensure airway patency during resuscitation. Therefore, the goal of our study was to evaluate the relationship between degree of head tilt as measured by sagittal MRI and patency of the airway in a cohort of neonates and young infants who underwent MRI.

## Materials and Methods

We retrospectively studied the MR images of the airway of neonates (age: 0–28 days) and young infants (age: 29 days– 4 months) at our institution. The Johns Hopkins Institutional Review Board (IRB) approved the study. Since it was a retrospective review of MRI of patients in our Johns Hopkins Hospital, informed consent, written or oral, was NOT obtained from the participants. The Johns Hopkins IRB provided waiver of informed consent due to the retrospective nature of the study. The data reported in the manuscript were not analyzed anonymously.

### MRI acquisition

All MRI studies were performed on a 1.5 T scanner (Siemens Avanto, Erlangen, Germany) using our standard departmental protocol including 3D-T1- and axial T2-weighted images, an axial fluid attenuation inversion recovery (FLAIR) sequence, and an axial diffusion tensor imaging (DTI) sequence with diffusion gradients along 20 non-collinear directions. For the acquisition of high-resolution axial T2-weighted images, the following parameters were used: repetition time (TR) 4190 ms, echo time (TE) 104 ms, slice thickness 4.0 mm, field-of-view (FOV) 200x200 mm, matrix size 320x320.

### Measurements

**Fig 1:**
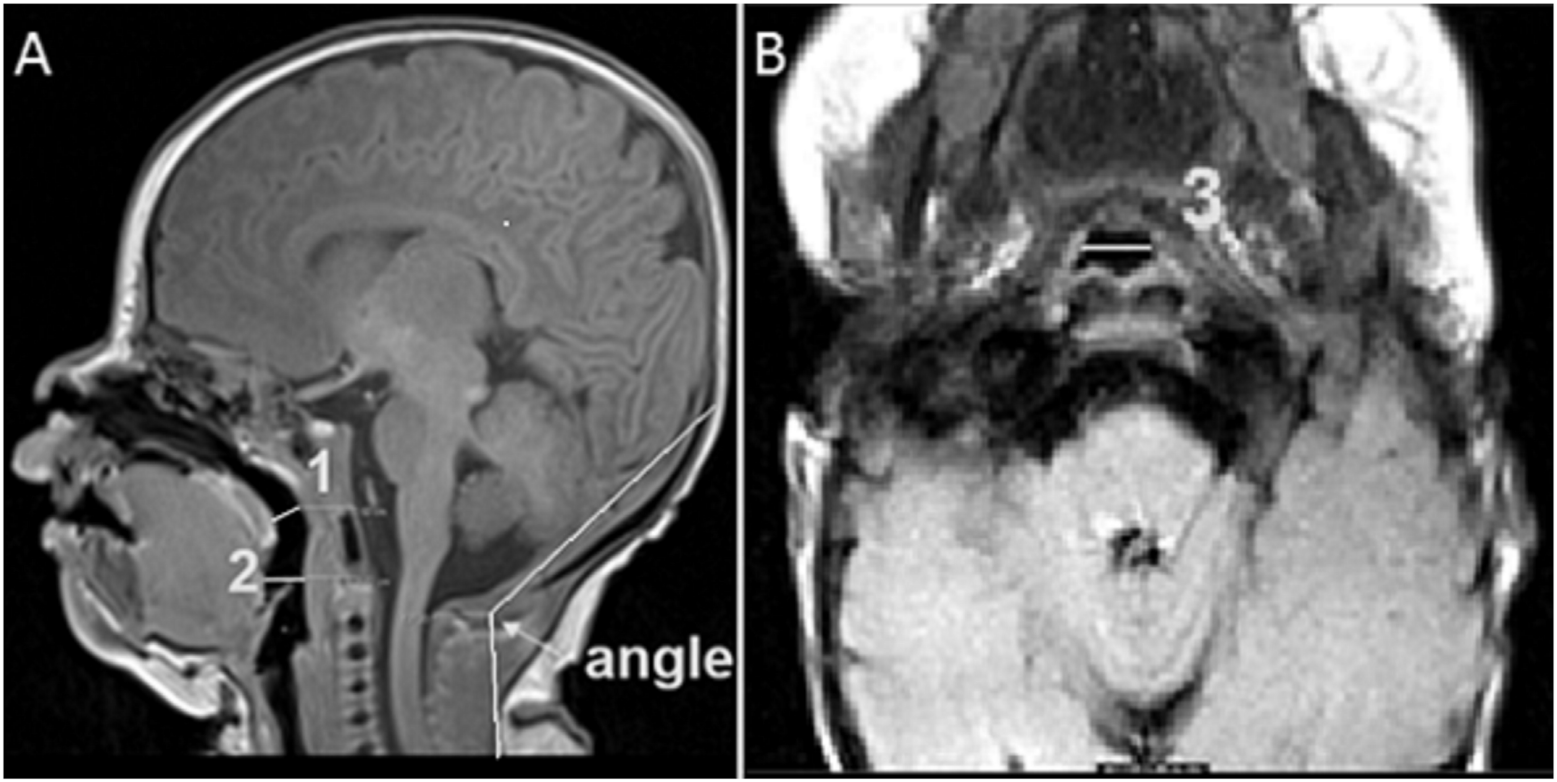
A—T1-weighted MR image of head-neck region of a newborn infant showing head-tilt angle between the occipito-ophisthion-C7 spinous process, AP diameters of airway at the level of palate (1) and tongue (2) in mid-sagittal plane. B—Axial T1-weighted MR image of head-neck region of a newborn infant showing lateral diameter of airway at the tongue level (3).

In this retrospective study, a total of 827 children between 0–4 months of age who underwent MRI of head and neck at Johns Hopkins Hospital between January 1984 and December 2013 were identified. Of these, 760 subjects were excluded due to either an artificial airway or a distorted airway from malformation. A total of 63 children (53 neonates and 14 infants) were included for measurements and analysis. MRI records were evaluated for any reported artificial airway and/or airway malformation. If the MRI records did not report presence of an artificial airway, we evaluated the sagittal and axial MRI images for any artificial airway. All the images were reviewed by our pediatric radiologists for confirmation of presence of any artificial airway and/or airway malformation for exclusion from the analysis. On the remaining patients, the sagittal and axial MRI images were assessed for airway patency. The antero-posterior (AP) and lateral airway diameters were measured at the level of palate and dorsum of tongue. We defined a blocked airway as an airway with an immeasurable AP and/or lateral diameter on MRI imaging. We defined head-tilt angle a priori as an angle between the occipito-ophisthion line and the ophisthion-C7 spine process line on a midline sagittal MR image (Fig 1). We evaluated medical records of those newborn infants who were included in the study for age, sex, weight, gestational age, Apgar scores at birth, features suggestive of hypoxic-ischemic encephalopathy (HIE), exposure to central nervous system (CNS) depressants like antiepileptic drugs (AEDs), and/or sedatives around the time of MRI. Newborn infants unconscious from birth asphyxia with very low Apgar scores have a tendency for airway obstruction due to loss of airway tone and glossoptosis. Similarly, spontaneously breathing newborn infants in our study with CNS depression due to either HIE and/or exposure to AEDs and sedatives were at a risk of airway compromise due to relaxation of airway tone and glossoptosis. We, therefore, performed analysis of head-tilt angle in relation to airway size in those newborn infants who had CNS depression from HIE and/or exposure to CNS depressant agents. We compared the head-tilt angle (median ± SD) of patent airway versus blocked airway. We analyzed data for any correlation between median head-tilt angle of airway patency and age, gestational age and weight of the newborn infant.

### Statistical analysis

We used a Mann-Whitney U test to compare the blocked airway to the patent airway instead of a standard student’s t-test (p < 0.05 as significant). We performed logistic regression analysis of proportion of patent airways over different head-tilt angles. We calculated a Pearson correlation coefficient for assessing correlation of the age, gestational age, and weight of the patient in comparison to the head-tilt angle for airway patency.

## Results

**Fig 2:**
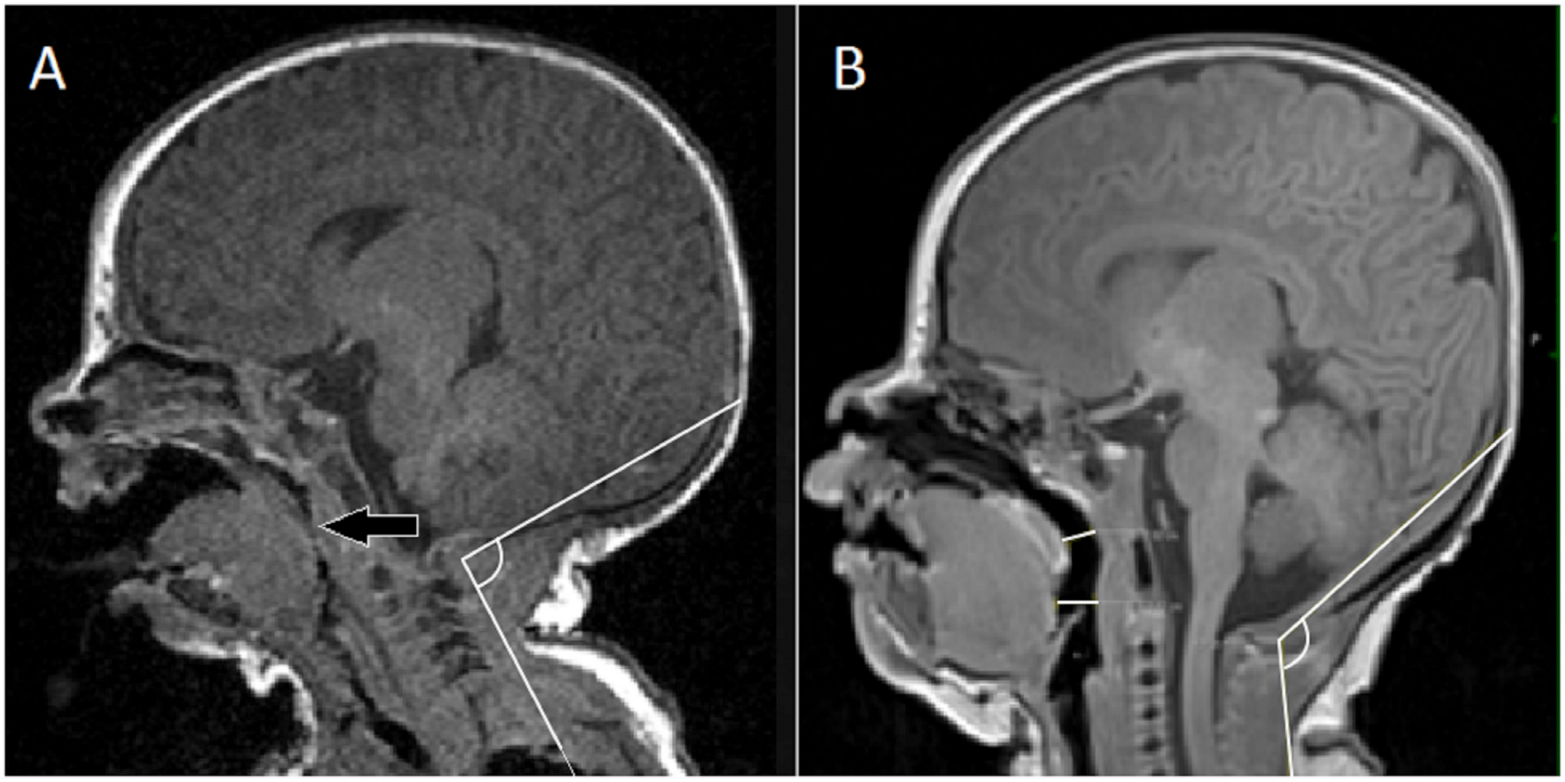
A—Sagittal T1-weighted MR image of head-neck region of a newborn-infant showing obstructed airway at the tongue level. B—Sagittal T1-weighted MR image of head-neck region of a newborn-infant showing a patent airway.

**Fig 3:**
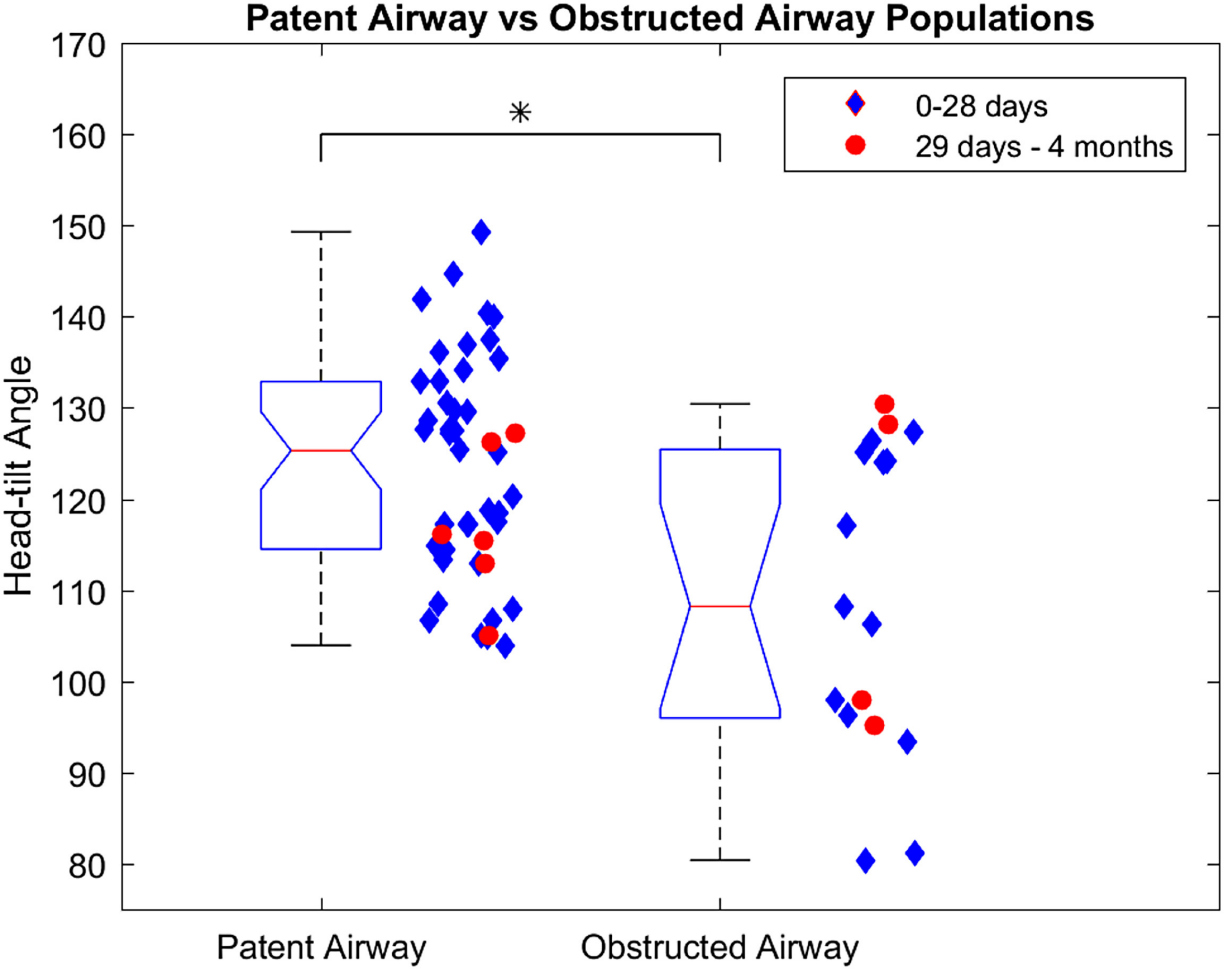
Box-plot diagram of head-tilt angle of patent airway as compared to that of obstructed airway. The median head-tilt angle associated with patent airway (125.3° ± 11.9°) was significantly different from median head-tilt angle (108.2° ± 17.1°) associated with an obstructed airway (p = 0.0045).

**Fig 4:**
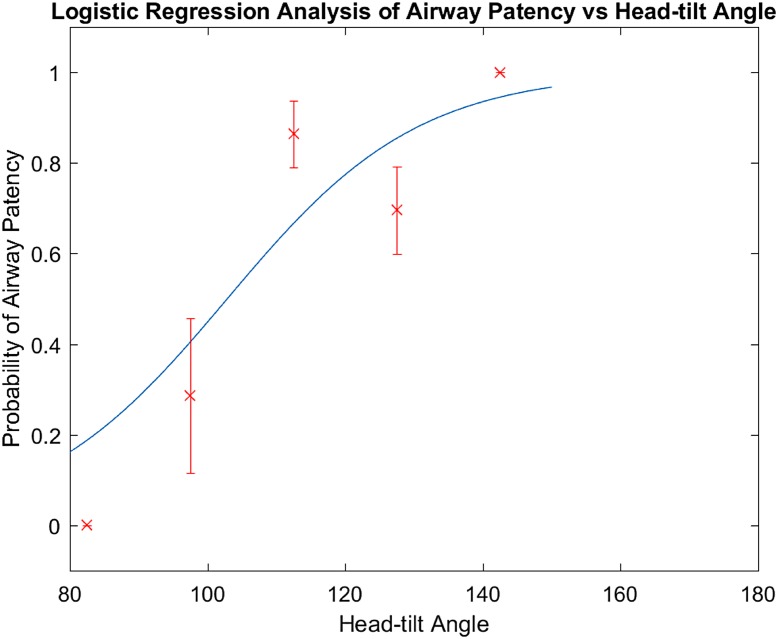
Logistic regression analysis of airway patency versus head-tilt angle shows that the probability of a patent airway progressively increases with increasing head-tilt angle. There is at least a 95% probability that an airway will be patent between head-tilt angle 144–150°. Y-axis represents the proportion of patent airways with standard error of proportion and X-axis represents 15° bins of head-tilt angles.

Of the 63 children who were analyzed, 17 had evidence of airway obstruction, 46 had a patent airway on MRI. An obstructed airway was visible as complete occlusion of airway lumen at the level of palate and/or dorsum of tongue (Fig 2). Also, 16/63 had underlying HIE and 47/63 newborn infants had exposure to sedative medications during MRI. A one-sample Kolmogorov-Smirnov test confirmed that the samples were not normally distributed with respect to head-tilt angle. Therefore, we used a Mann-Whitney U test to compare the blocked airway to the patent airway instead of a standard student’s t-test (p < 0.05 as significant). In spontaneously breathing and neurologically depressed newborn infants, the median head-tilt angle associated with patent airway (125.3° ± 11.9°) was significantly different from median head-tilt angle (108.2° ± 17.1°) associated with a blocked airway (p = 0.0045) (Fig 3). The airway diameters (mean ± SD) in spontaneously breathing, sedated children with open airway were 5.8 ± 1.8 mm (AP at palate), 6.3 ± 1.5 mm (AP at dorsum of tongue) and 7.4 ± 2.7 mm (lateral). The Pearson correlation coefficient did not show any correlation between median head-tilt angle of airway patency and either age, gestational age, and weight of the newborn infant (Table 1). The logistic regression model showed that the proportion of patent airways progressively increased with an increasing head-tilt angle (Table 2). There was at least a 95% probability that an airway will be patent between head-tilt angle 144–150° (Fig 4). Pearson's Chi-square test (p = 0.440), deviance test (p = 0.441), and Hosmer-Lemeshow test (p = 0.409) confirmed the goodness-of-fit of the logistic regression model.

**Table 1:** Correlation between Head-tilt Angle of Patent Airways and Age, Weight, and Gestational Age of Newborn Infants.

Variable	Correlation Coefficient	p-value*
Age (days)	-0.134	0.294
Weight (kg)	-0.065	0.609
Gestational Age (weeks)	0.057	0.659

*Pearson correlation

**Table 2:** Logistic Regression Model of Patent Airway vs Head-tilt Angle.

Variables	Beta	Odds Ratio	95% CI	p value
Head-tilt angle	0.0719	1.0745	1.029,1.132	0.002

## Discussion

Airway patency is a cornerstone of neonatal resuscitation. Various head positions and airway maneuvers are described to maintain airway patency during resuscitation in newborn infants. Neither sniffing position (recommended by NRP) nor neutral head position (recommended by NLS) have been evaluated for airway patency in newborns and infants [12]. We hereby describe the MRI findings of airway patency in relation to head tilt position. To our knowledge, this is the first study of quantitative analysis of head-tilt position and airway patency in neurologically depressed neonates and young infants using MRI images. The study findings are clinically relevant for several reasons. It clarifies an unanswered question about head tilt position for airway resuscitation in neonates and young infants and it identifies a specific angle of head-tilt position for future development of a resuscitation device. There are several common problems that interfere with effective neonatal resuscitation, one of which is improper head and neck position [13]. Airway occlusion due to improper head-neck position within the golden minute of neonatal resuscitation potentially contributes to increased neonatal mortality. In several neonatal resuscitation settings, especially in the developing countries, a semi-trained birth attendant may have to independently perform the complex steps of neonatal resuscitation. In such a situation, the complex resuscitation could be simplified by using a device that provides support for the head-tilt position as defined by our study.

In 1950s, Safar et al described a pioneering study of airway patency in relation to head position [6,7]. However, the study did not include children and did not provide any quantitative information on head-tilt position for airway patency. In a study of airway patency in infants who died of sudden infant death syndrome (SIDS), neutral and extended neck positions were determined to be better than the flexed position for air entry to the trachea [14]. Similarly, in a post-mortem study of infant airways, head flexion was associated with elevated upper airway closing pressure and tendency for upper airway collapse, whereas head extension was associated with decrease in upper airway closing pressure and patent upper airway [15]. Both studies were done under non-physiologic, post-mortem conditions, in absence of any spontaneous breathing and it is likely that rigor mortis confounded the study findings.

Our study evaluated airways in spontaneously breathing, sedated or obtunded neonates and young infants, which represents a more physiologic approach for defining head-tilt position for airway patency. In previous studies, the angle between a line from the outer canthus to the external auditory meatus and the longitudinal axis of the infant’s trunk was evaluated as the neck angle for airway patency [9,15]. Since our goal was to define the angle or range of the head-tilt position that would be associated with a patent airway and could be potentially used for creating an adjustable neck/shoulder roll for resuscitation of newborn infants, we defined head-tilt angle a priori as an angle between the occiput-ophisthion-C7 spine process on the mid-sagittal head and neck MR image (Fig 1). The median head-tilt angle, which was associated with open airway in newborns and infants, was found to be 125.3° ± 11.9° in our study. Furthermore, the logistic regression analysis indicates that the probability of having a patent airway increases progressively with increasing head-tilt angle, with >95% probability of airway patency at head-tilt angles 144–150° (slightly extended head position) and <20% probability of a patent airway at airway angles < 90° (hyper-extended head position). The higher probability of airway occlusion in a hyperextended head position could be explained from a higher probability of backward movement of tongue and/or palate against the posterior pharyngeal wall due to effect of gravity. Our findings support the findings of other studies that concluded that slightly extended head position is likely to be associated with a patent airway in neonates. Our findings are consistent with NRP recommendations of a slightly extended head position during neonatal resuscitation. This is the first evidence based study using MRI to define head-tilt position for airway patency to enable use of the data for creating a device to allow an open airway during resuscitation or procedural sedation in newborn infants.

The study limitations are the retrospective study design using static MRI images, the small sample size, and exclusion of neonatal infants with airway malformations. The static MRI images used in the study to determine the relation between airway diameters and head positions may not correlate with dynamic airway changes occurring during the respiratory cycle and at different levels of sedation. A prospective dynamic airway study using different head positions combined with multi-modality monitoring like end-tidal capnography and tidal volume measurements may give more detailed answer. Because of the retrospective nature of the study, 3D analysis of the anatomy was not feasible. In addition, infants with airway malformations were excluded. In our study, it is unknown whether a shoulder or neck roll was used in neonates and infants during the MRI to maintain airway patency. However, since the head-tilt angle was measured using bony landmarks at the nape of the neck, use of shoulder or neck roll should not affect our findings of correlation of head-tilt angle with airway patency.

## Conclusions

There is a >95% probability of a patent airway at a head-tilt angle (between occiput-ophisthion-cervical spine) of 144–150° in spontaneously breathing, sedated children between the ages of 0–4 months. The head-tilt angle range of 144–150° for airway patency corresponds to a slightly extended head position.
